# Supplementary material for: Transcriptional analysis in multiple barley varieties identifies signatures of waterlogging response
Source: Plant Direct. 2023 Aug 12;7(8):e518. doi: 10.1002/pld3.518 (PMC10422865; doi:10.1002/pld3.518)
Supplement: Supplementary file 1 — Table S1: List of varieties used in this study. All varieties listed are winter varieties, except for Golden Promise, which is a spring variety. Apart from Infinity and Golden Promise, all varieties originated from the AGOUEB population (Thomas et al., 2014). Table S2: List of oligonucleotides used in this study. Figure S1: Selection of reference genes for RT‐qPCR analysis, and of time points for waterlogging experiments. (A) Cp values for selected reference genes. Whole roots were collected at different time‐points after the beginning of waterlogging treatment, and pooled for RNA extraction. Expression was determined using RT‐qPCR. Cp values are shown for three biological replicates using the Golden Promise variety. (B) Transcriptional response of selected hypoxia response marker genes at different time points after the beginning of waterlogging treatment. Mean values of 2 biological replicates are shown. Error bars indicate standard deviations. (C) Mean Cp values for HvACTIN for all varieties and RT‐qPCRs presented in Figure 1. Error bars correspond to SEM. Figure S2: Relative expression of HvPDC1 in selected varieties. HvPDC1 expression relative to ACTIN in untreated (open symbols; normal watering) and waterlogged (blue symbols) plants after 24 h of treatment. The dashed line corresponds to the average relative expression of HvPDC1 for waterlogged samples for all varieties tested across all replicates. Data shown is from three biological replicates, except Golden Promise (Golden P.) which had 4 biological replicates. Each biological replicate corresponds to whole roots from 3 plants pooled. Error bars correspond to SEM. See Figure 1D for the results of two‐way ANOVA. Figure S3: Assessing the quality of RNA‐seq datasets. (A) Details of samples used for RNASeq analysis. (B) Heatmap showing sample‐to‐sample Euclidean distance (generated by PCA explorer package in R). (C) Absolute number of reads aligned to Morex v3 for each sample. (D) PCA plot showing PC2 vs PC1 wi [file PLD3-7-e518-s007.pdf]

Transcriptional analysis in multiple barley varieties identifies signatures of waterlogging response

Alexandra Miricescu[1,2,#], Ailbhe Jane Brazel[1,#,*], Joseph Beegan[3], Frank Wellmer[3], and Emmanuelle Graciet[1,4,*]

[1] Maynooth University, Department of Biology, Maynooth, Co. Kildare, Ireland

[2] Current address: Pesticide Registration Division, Department of Agriculture, Food and the Marine, Backweston Campus, Celbridge, Co. Kildare, W23X3PH, Ireland

[3] Smurfit Institute of Genetics, Trinity College Dublin, Dublin, Ireland

[4] Kathleen Lonsdale Institute for Human Health Research, Maynooth University, Maynooth, Co. Kildare, Ireland

Supplementary Tables

Supplementary Table S1: List of varieties used in this study. All varieties listed are winter varieties, except for *Golden Promise*, which is a spring variety. Apart from *Infinity* and *Golden Promise*, all varieties originated from the AGOUEB population (Thomas *et al.*, 2014).

**Variety name**	**Row number**
Golden Promise	2-row
Infinity	2-row
Passport	6-row
Pilastro	6-row
Regina	2-row
Arma	6-row
Isa	6-row
Louise	2-row
Retriever	2-row
Dura	6-row
Masquerade	2-row
Madrigal	2-row
Vesuvius	2-row
Tapir	6-row
Kosmos	6-row
Maeva	6-row
Siberia	6-row
Breeze	2-row
Cavalier	2-row
Mahogany	2-row
Tamaris	6-row

Supplementary Table S2: List of oligonucleotides used in this study.

Oligo name	Gene description	Gene ID	Oligonucleotide sequence (5' -> 3')	Reference
AM45	HvACTIN	HORVU1Hr1G002840	GCAAGTGGTCGTACTACTGGTATCGTTC	(Miricescu *et al.*, 2021)
AM46	HvACTIN	HORVU1Hr1G002840	GGATCTTCATAAGGGAGTCCGTGAGAT	(Miricescu *et al.*, 2021)
AM8	HvTUBULIN	HORVU.MOREX.r3.1HG0082050	TGGTCATTACACCATTGGCAAGGAGA	This study
AM9	HvTUBULIN	HORVU.MOREX.r3.1HG0082050	GTGTATGTTGGGCGCTCAATGTCA	This study
AM49	HvGAPDH	HORVU7Hr1G074690	GCTCACTTGAAGGGTGGTGCC	This study
AM50	HvGAPDH	HORVU7Hr1G074690	TGATGGCATGAACAGTGGTCATCAGAC	This study
HSP70F	HvHSP70	HORVU.MOREX.r3.5HG0526250	GCTCAACATGGACCTCTTCAGG	(Walling *et al.*, 2018)
HSP70R	HvHSP70	HORVU.MOREX.r3.5HG0526250	CCGACAAGGACAACATCATGG	(Walling *et al.*, 2018)
AM47	HvADP	HORVU3Hr1G079700	CCACCATCCCAACCATCGGTTT	This study
AM48	HvADP	HORVU3Hr1G079700	CCTGCGTATTCTGGAAGTAGTGCCT	This study
HvADH1F	HvADH1	HORVU4Hr1G016810	CACTGACCTGCCCAATGTC	(Mendiondo *et al.*, 2016)
HvADH1R	HvADH1	HORVU4Hr1G016810	GCACGCTGTGTGTGATGAA	(Mendiondo *et al.*, 2016)
AM51	HvHB	HORVU4Hr1G066200	CGGGAAGGAAGCCATGTCTGC	(Miricescu *et al.*, 2021)
AM52	HvHB	HORVU4Hr1G066200	TCTGCCTCGCCGACGG	(Miricescu *et al.*, 2021)
AM53	HvPDC	HORVU4Hr1G056050	CCGCCTACGAGAACTACAAGAGGATC	This study
AM54	HvPDC	HORVU4Hr1G056050	TTCATACCCGCAGCCCTCG	This study

Supplementary Figures

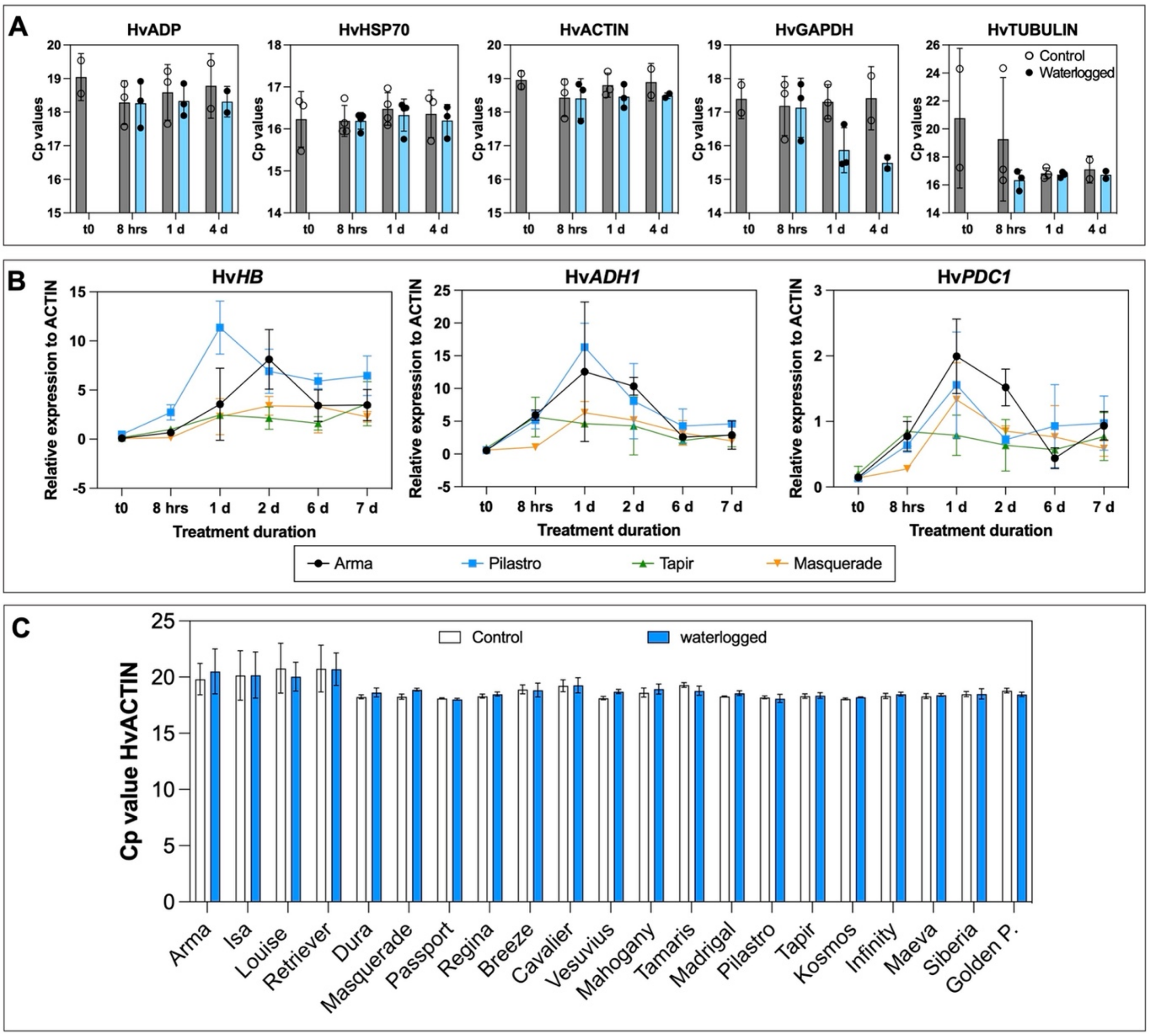

Figure S1: Selection of reference genes for RT-qPCR analysis, and of time points for waterlogging experiments. (A) Cp values for selected reference genes. Whole roots were collected at different time-points after the beginning of waterlogging treatment, and pooled for RNA extraction. Expression was determined using RT-qPCR. Cp values are shown for three biological replicates using the *Golden Promise* variety. (B) Transcriptional response of selected hypoxia response marker genes at different time points after the beginning of waterlogging treatment. Mean values of 2 biological replicates are shown. Error bars indicate standard deviations. (C) Mean Cp values for Hv*ACTIN* for all varieties and RT-qPCRs presented in Fig. 1. Error bars correspond to SEM.

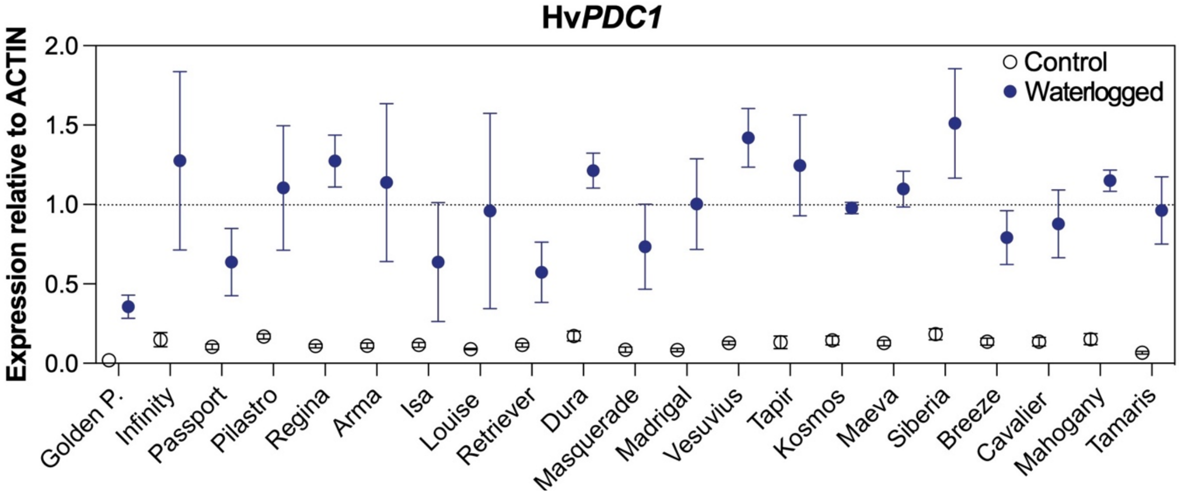

Figure S2: Relative expression of Hv*PDC1* in selected varieties. Hv*PDC1* expression relative to *ACTIN* in untreated (open symbols; normal watering) and waterlogged (blue symbols) plants after 24 h of treatment. The dashed line corresponds to the average relative expression of Hv*PDC1* for waterlogged samples for all varieties tested across all replicates. Data shown is from three biological replicates, except *Golden Promise* (*Golden P.*) which had 4 biological replicates. Each biological replicate corresponds to whole roots from 3 plants pooled. Error bars correspond to SEM. See Figure 1D for the results of two-way ANOVA.

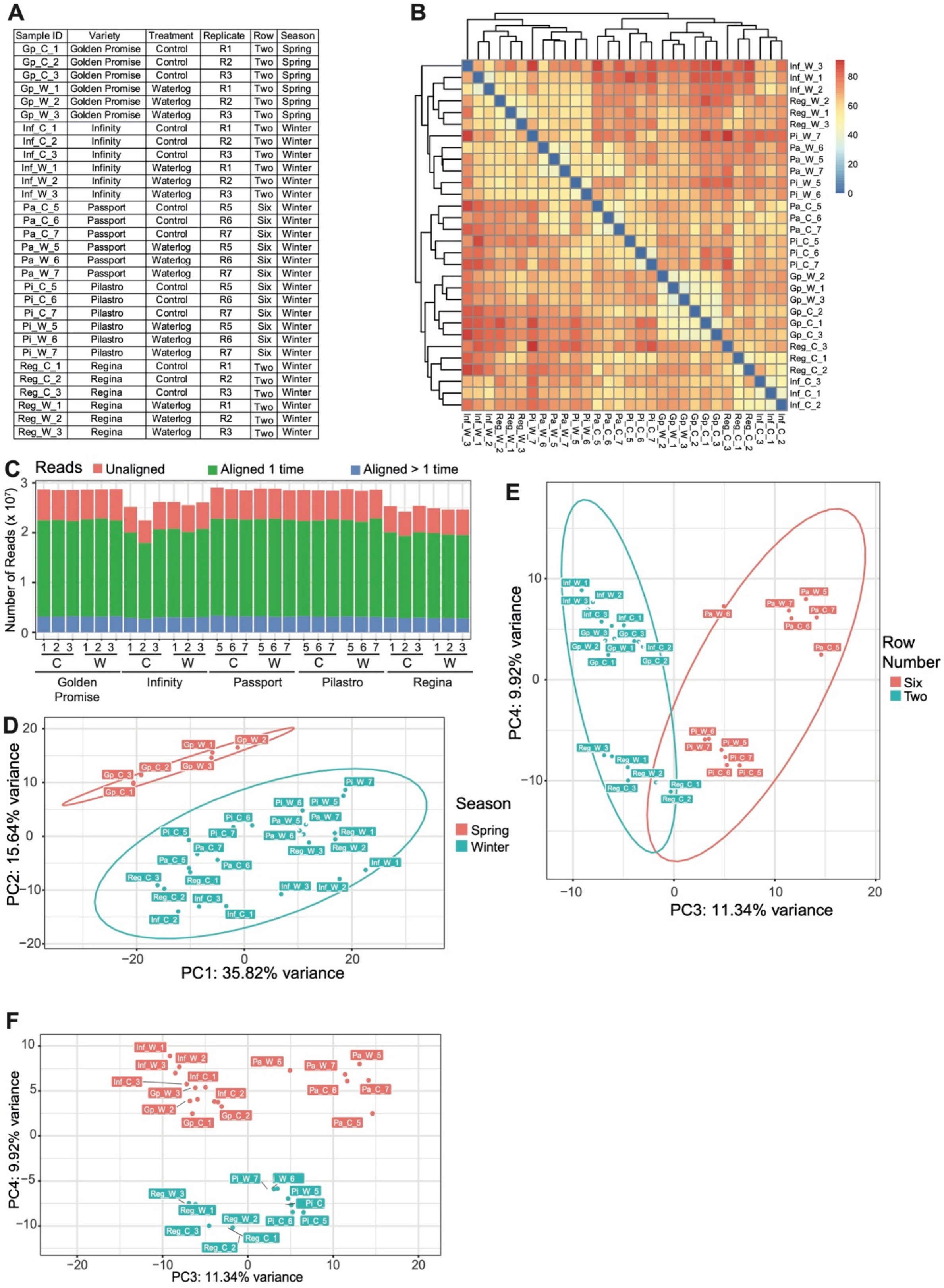

Sample ID	Variety	Treatment	Replicate	Row	Season
Gp_C_1	Golden Promise	Control	R1	Two	Spring
Gp_C_2	Golden Promise	Control	R2	Two	Spring
Gp_C_3	Golden Promise	Control	R3	Two	Spring
Gp_W_1	Golden Promise	Waterlog	R1	Two	Spring
Gp_W_2	Golden Promise	Waterlog	R2	Two	Spring
Gp_W_3	Golden Promise	Waterlog	R3	Two	Spring
Inf_C_1	Infinity	Control	R1	Two	Winter
Inf_C_2	Infinity	Control	R2	Two	Winter
Inf_C_3	Infinity	Control	R3	Two	Winter
Inf_W_1	Infinity	Waterlog	R1	Two	Winter
Inf_W_2	Infinity	Waterlog	R2	Two	Winter
Inf_W_3	Infinity	Waterlog	R3	Two	Winter
Pa_C_5	Passport	Control	R5	Six	Winter
Pa_C_6	Passport	Control	R6	Six	Winter
Pa_C_7	Passport	Control	R7	Six	Winter
Pa_W_5	Passport	Waterlog	R5	Six	Winter
Pa_W_6	Passport	Waterlog	R6	Six	Winter
Pa_W_7	Passport	Waterlog	R7	Six	Winter
Pi_C_5	Pilastro	Control	R5	Six	Winter
Pi_C_6	Pilastro	Control	R6	Six	Winter
Pi_C_7	Pilastro	Control	R7	Six	Winter
Pi_W_5	Pilastro	Waterlog	R5	Six	Winter
Pi_W_6	Pilastro	Waterlog	R6	Six	Winter
Pi_W_7	Pilastro	Waterlog	R7	Six	Winter
Reg_C_1	Regina	Control	R1	Two	Winter
Reg_C_2	Regina	Control	R2	Two	Winter
Reg_C_3	Regina	Control	R3	Two	Winter
Reg_W_1	Regina	Waterlog	R1	Two	Winter
Reg_W_2	Regina	Waterlog	R2	Two	Winter
Reg_W_3	Regina	Waterlog	R3	Two	Winter

Figure S3: Assessing the quality of RNA-seq datasets. (A) Details of samples used for RNA-Seq analysis. (B) Heatmap showing sample-to-sample Euclidean distance (generated by *PCA explorer* package in *R*). (C) Absolute number of reads aligned to Morex v3 for each sample. (D) PCA plot showing PC2 vs PC1 with ellipses grouping samples based on their sowing season. Plot is identical to Figure 3A, but highlights grouping based on PC2 instead of PC1.

(E) PCA plot showing PC4 vs PC3 with ellipses grouping by row number. (F) PCA plot showing PC4 vs PC3. Plot is identical to (E), but colour grouping based on PC4 instead of PC3.

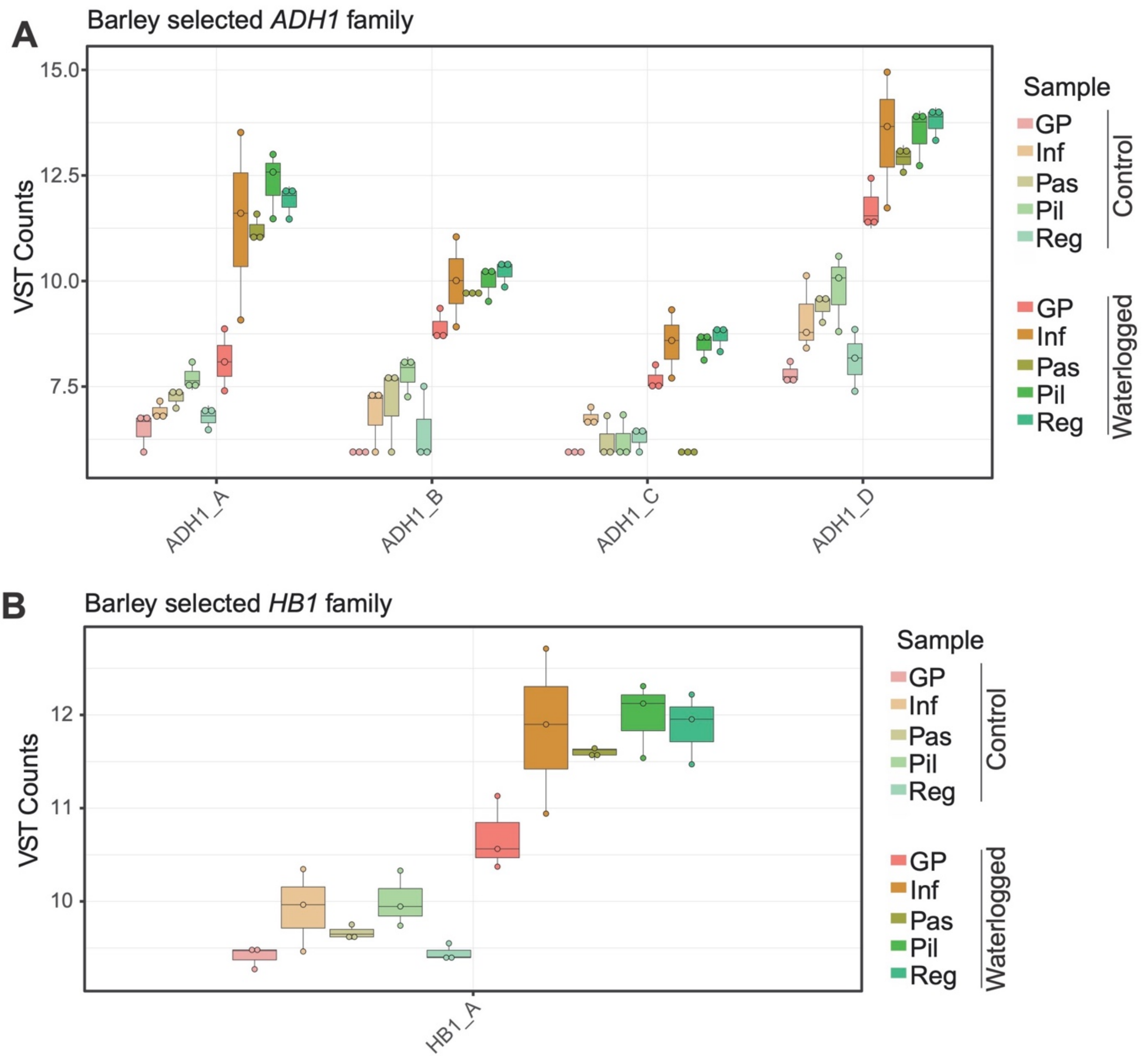

Figure S4: Internal validation of RNA-seq datasets. (A) Boxplots showing variance stabilised transformed (VST) read counts for barley orthologs of Arabidopsis *ADH1* that responded to waterlogging treatment: ADH1_A (HORVU.MOREX.r3.1HG0082960), ADH1_B (HORVU.MOREX.r3.2HG0115170), ADH1_C (HORVU.MOREX.r3.3HG0252910) and ADH1_D (HORVU.MOREX.r3.4HG0345740). (B) Boxplots showing VST read counts for a barley ortholog of Arabidopsis HB1 that responded to waterlogging treatment (HB1_A (HORVU.MOREX.r3.7HG0715010)). In (A) and (B), VST counts from control and waterlogged samples for *Golden Promise* (GP), *Infinity* (Inf), *Passport* (Pas), *Pilastro* (Pil) and *Regina* (Reg.) are shown.

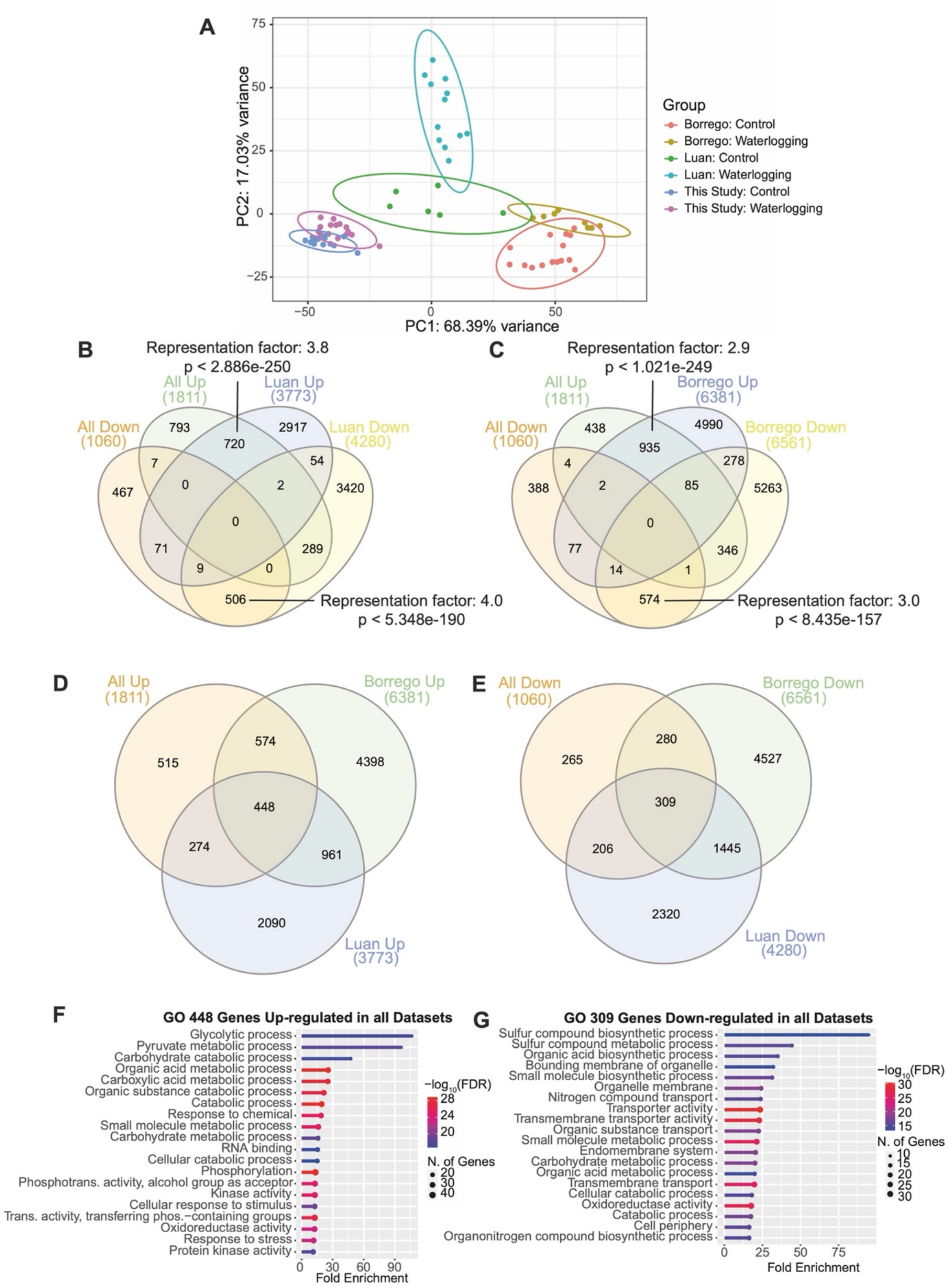

Figure S5: Comparison of RNA-seq datasets with published datasets. (A) PCA plot showing PC1 vs PC2 with ellipses grouping samples based on the dataset origin and treatment. (B) Overlap of DEGs identified in this study and those determined after re-analysis of raw data from Luan *et al.* (Luan *et al.*, 2022) (adjusted *p*-value < 0.05). (C) Overlap of DEGs identified

in this study and those determined after re-analysis of raw data from Borrego-Benjumea *et al*. (Borrego-Benjumea *et al.*, 2020) (adjusted *p*-value < 0.05). (D) Overlap of up-regulated DEGs identified in this study with up-regulated DEGs from Luan et al. and Borrego-Benjumea et al. (E) Overlap of down-regulated DEGs identified in this study with down-regulated DEGs from Luan et al. and Borrego-Benjumea et al. (F) Top 20 GO terms enriched in 448 shared up-regulated DEGs identified in (D). (G) Top 20 GO terms enriched in 309 shared up-regulated DEGs identified in (E).

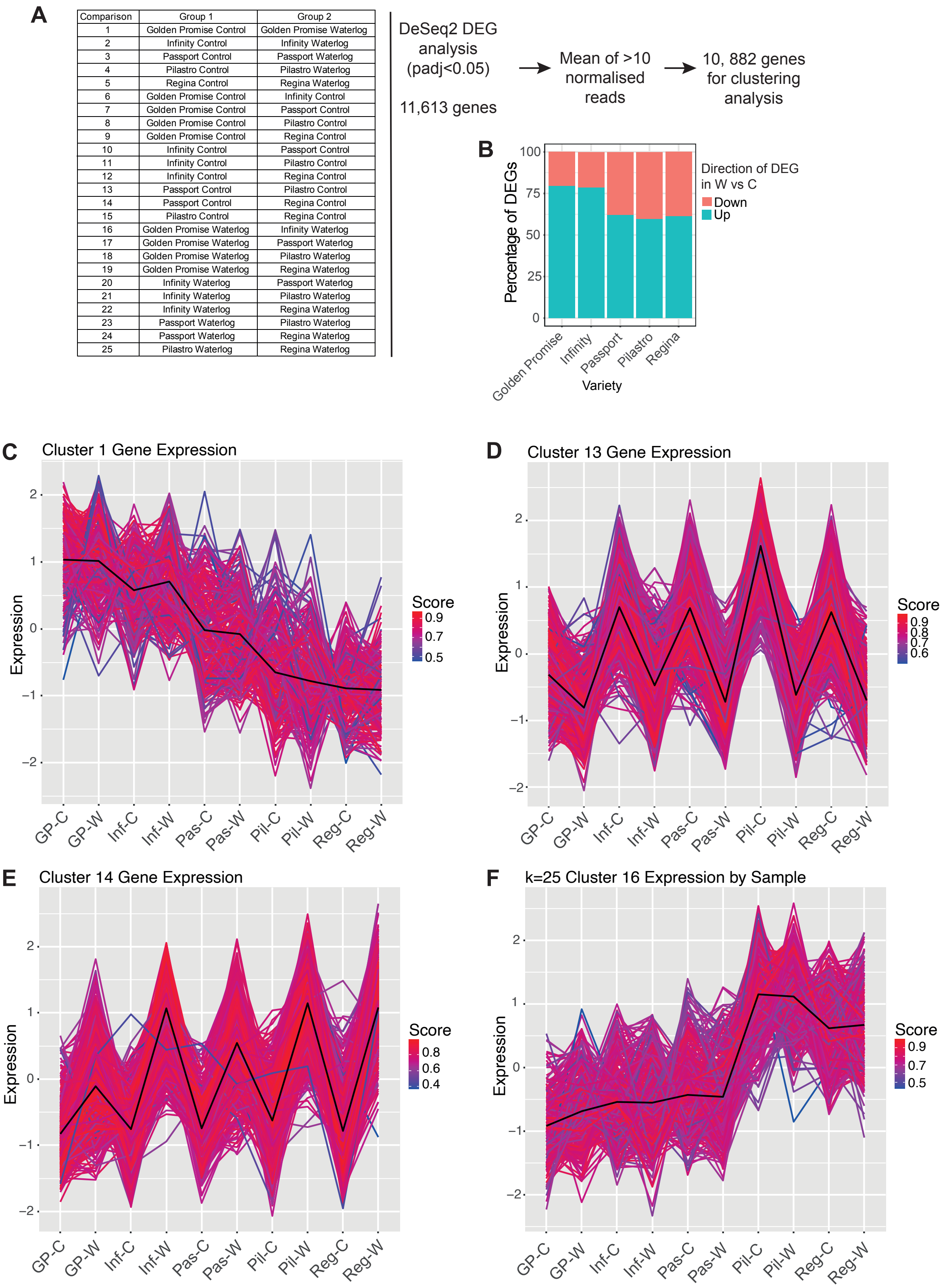

Comparison	Group 1	Group 2
1	Golden Promise Control	Golden Promise Waterlog
2	Infinity Control	Infinity Waterlog
3	Passport Control	Passport Waterlog
4	Pilastro Control	Pilastro Waterlog
5	Regina Control	Regina Waterlog
6	Golden Promise Control	Infinity Control
7	Golden Promise Control	Passport Control
8	Golden Promise Control	Pilastro Control
9	Golden Promise Control	Regina Control
10	Infinity Control	Passport Control
11	Infinity Control	Pilastro Control
12	Infinity Control	Regina Control
13	Passport Control	Pilastro Control
14	Passport Control	Regina Control
15	Pilastro Control	Regina Control
16	Golden Promise Waterlog	Infinity Waterlog
17	Golden Promise Waterlog	Passport Waterlog
18	Golden Promise Waterlog	Pilastro Waterlog
19	Golden Promise Waterlog	Regina Waterlog
20	Infinity Waterlog	Passport Waterlog
21	Infinity Waterlog	Pilastro Waterlog
22	Infinity Waterlog	Regina Waterlog
23	Passport Waterlog	Pilastro Waterlog
24	Passport Waterlog	Regina Waterlog
25	Pilastro Waterlog	Regina Waterlog

Figure S6: Clustering Analysis. (A) Structure of data used to generate DEG list used for *k*-means clustering analysis. The means of normalized read counts from 3 biological replicates generated from *DeSeq2* were filtered as follows. *Deseq2* DEG analysis was performed and results for 25 comparisons were extracted. Firstly, a list of all 11,613 differentially expressed

genes (DEGs) filtered by adjusted *p*-value < 0.05 was generated by combining DEGs from waterlogged *vs* control samples from the same variety and DEGs from pairwise comparisons of each control to each other control variety and each waterlogged to each other waterlogged variety (25 DEG lists). Next the DEGs with a mean of <10 normalised reads were removed, leaving 10,882 genes for clustering analysis. (B) Number of up and down-regulated DEGs (filtered by adjusted *p*-value < 0.05) in waterlogged *vs* control samples for each variety as a percentage of all the DEGs in that sample. Gene expression patterns are shown for DEGs from cluster 1 (C), cluster 13 (D), cluster 14 (E) and cluster 16 (F). The expression of the core centroid is shown in black while the expression of each gene within the cluster is shown in colour. The score indicates the correlation of expression of each gene within a cluster to the expression pattern of the core centroid of that cluster. Scores closer to 1 indicate a better fit to the centroid expression pattern. Data is shown for control (C) and waterlogged (W) samples from *Golden Promise* (GP), *Infinity* (Inf), *Passport* (Pas), *Pilastro* (Pil) and *Regina* (Reg).

References

Borrego-Benjumea A, Carter A, Tucker JR, Yao Z, Xu W, Badea A. 2020. Genome-Wide Analysis of Gene Expression Provides New Insights into Waterlogging Responses in Barley (Hordeum vulgare L.). Plants (Basel) **9**, 240.
Luan H, Chen C, Yang J, Qiao H, Li H, Li S, Zheng J, Shen H, Xu X, Wang J. 2022. Genome-wide association scan and transcriptome analysis reveal candidate genes for waterlogging tolerance in cultivated barley. Front Plant Sci **13**, 1048939.
Mendiondo GM, Gibbs DJ, Szurman-Zubrzycka M, Korn A, Marquez J, Szarejko I, Maluszynski M, King J, Axcell B, Smart K, Corbineau F, Holdsworth MJ. 2016. Enhanced waterlogging tolerance in barley by manipulation of expression of the N-end rule pathway E3 ligase PROTEOLYSIS6. Plant Biotechnology Journal **14**, 40-50.
Miricescu A, Byrne T, Doorly CM, Ng CKY, Barth S, Graciet E. 2021. Experimental comparison of two methods to study barley responses to partial submergence. Plant Methods **17**, 40.
Thomas W, Comadran J, Ramsay L, Shaw P, Marshall D, Newton A, O'Sullivan D, Cockram J, Mackay I, Bayles R, White J, Kearsey M, Luo Z, Wang M, Tapsell C, Harrap D, Werner P, Klose S, Bury P, Wroth J, Argilier O, Habgood R, Glew M, Bochard A-M, Gymer P, Vequaud D, Christerson T, Allvin B, Davies N, Broadbent R, Brosnan J, Bringhurst T, Booer C, Waugh R. 2014. Association genetics of UK elite barley (AGOUEB). Final Project Report to HGCA, No 528, 125pp.
Walling JG, Zalapa LA, Vinje MA. 2018. Evaluation and selection of internal reference genes from two- and six-row U.S. malting barley varieties throughout micromalting for use in RT-qPCR. PLoS One **13**, e0196966.
