# Supplementary material for: Transcriptional analysis in multiple barley varieties identifies signatures of waterlogging response
Source: Plant Direct. 2023 Aug 12;7(8):e518. doi: 10.1002/pld3.518 (PMC10422865; doi:10.1002/pld3.518)
Supplement: Supplementary file 7 — Supporting info item [file PLD3-7-e518-s005.docx]

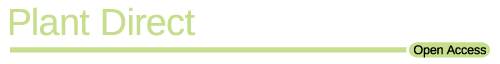


**DECISION LETTER- Round 1**

| July 14, 2023  Dr. Emmanuelle Graciet University of Ireland Maynooth Maynooth, N/A Ireland   RE: Transcriptional analysis in multiple barley varieties identifies signatures of waterlogging response  Dear Dr. Graciet:  Thank you for submitting to Plant Direct. All required reviews have been returned and we have now finished our evaluation of your manuscript. In light of the reviewers' and editor's comments, further revisions are needed before the paper can be accepted for publication in Plant Direct.  Please view the editors' and reviewers' comments below and use their suggestions as a guide while you work on your revision.  When uploading the revised version of this article, please be sure to include the following:  -A word document that contains your response to the reviewers. You should respond to each reviewer comment and note the changes made to the manuscript. If you do not agree with a reviewer's comment and choose not to make a suggested revision, please explain why. Please try to provide as complete an answer as possible to each reviewer's criticisms in the "response to reviewers" section. -A tracked changes document - A clean version of the latest version of the manuscript  To upload your revision, please click the link below. https://plantdirect.msubmit.net/cgi-bin/main.plex?el=A4Lr4bu6A7lpO4I3A9ftdHY9iDKKcPD6ajHrb6tdzAZ  In order to provide as timely a service as possible, we ask that your revision is resubmitted within three months after receipt of this request. If an extension is needed, please send a request, along with a brief explanation, to the editorial office at plantdirect@wiley.com .  Thank you very much for giving us an opportunity to review your work. I look forward to receiving the next version.   Sincerely, Ana Fortes   ---------------------------------------------------------------------------- Editor comments: One of the reviewers raised important aspects regarding experimental design that should be fully addressed/ justified ( e.g. number of biological replicates used in transcriptomics analyses.)  ---------------------------------------------------------------------------- Reviewer comments: Reviewer #1:  The manuscript titled "Transcriptional analysis in multiple barley varieties identifies signatures of waterlogging response" is very well-written with excellent description of the methods utilized in the study. Although similar work has been done previously, this study validates and extends previous work and includes characterization of the response of key hypoxia-induced genes that may suggest tolerance/ sensitivity of specific barley varieties as well as indicates commonalities amongst monocot and dicot response to hypoxia. Although the overall study is very good, biological representation needs better description in the methods section. The authors need to clarify what they mean when they state 'independent replicates' vs. technical and biological replicates. This was done in figure descriptions but should be clearly described in the methods. Minor concerns: Line 204: Please note the company or organization used to obtain Golden Promise seeds or make it clear that this variety came from the same source as the others. Line 262: Was only a single replicate tested for NGS? The authors state that the roots of three different biological replicates were pooled. This would result in a strong single pool but only a single biological replicate. Was this pooling then repeated three times for additional biological replication? Please clarify. Line 301: Missing year for citation. Line 304: Missing year for citation. Line 633: Missing page number. Line 649: Missing page number. Line 659: Missing page number. Line 670: Missing page number. Line 680: Missing page number. Line 733: Missing page number. Line 739: Bold volume number. Line 772: Missing page number. Figure 2: What do you mean by "independent replicates" here? Did you pool samples for measurements or average data from all roots/ shoots of each sample? Figure 5A: This figure could be improved by adding the gene names beside the heat map. This is cumbersome but very useful to decipher the exact genes altered in each group. The first citation for Supplemental Data is missing the page number and the last citation uses a slightly different format that the rest. Please correct.  Reviewer #2:  Alexandra Miricescu and coauthors set out to identify variety-dependent and variety-independent transcriptome signatures in response to waterlogging in barley roots and link it to the plant physiological responses to the excess of water. The study follows a logical design, and all methods are up to scrutiny. Presented results support the conclusions in most of the cases. I have a couple of suggestion that might improve the current manuscript, mainly regarding methods description, and visual presentation of data.  1. HvACTIN was selected as reference gene based on its stable expression under control and waterlogged conditions over time. However, expression is shown only in one variety (Fig. S1)? Did authors make sure this gene is expressed to the same levels in few more varieties? I think it is essential to test it.  2. The method section should clarify number of replicates used for transcriptome analysis (considering that 3 root system were pooled as one replicate), as it is done in the figure 1  3. Figure 1 mentions: "Note: expression values for Infinity shown in (B) and (C) were published in (Miricescu et al., 2021)." Were the conditions of this study the same as presented here? If values for this variety comes from independent experiment, I would suggest to plot them separately. 4. Figure 2. I am again confused about the number of replicates. Do authors refer to independent experiments as independent replicates? The use of colors and shapes is not easy to follow. The shapes in the crowd of datapoints are hard to notice -I would suggest using two colors to mark control and waterlogging conditions. The colors indicating individual replicates (in my understanding independent experiments, but please clarify) are also hard to distinguish. If the authors aimed to test its effect I would rather suggest to analyze the data with a mixed model, where experimental trail is included as an independent factor.  5. In figure 3 I suggest using different colors and shadings (or other visualization) to indicate other variables, like variety, 2 vs 6 row and winter vs spring variety.  6. Figure 3C, Figure S4. Colors used are confusing, I would suggest using same color for each genotype and denote conditions by shading or intensity.  7. In line 422, the authors refer to several Arabidopsis homologs in barley. Is the function of the genes also conserved (are they orthologs?). Do authors have phylogenetic trees supporting this relationships?  8. For the heatmap in figure 5 I would suggest clustering the columns as well -this would allow to see responses of which varieties are similar. I would also love to see it with the relation to the phenotypic observations, maybe by highlighting Regina as the one with least response in root and shoot growth?  9.What was the p-value threshold fr the GO enrichment analysis?  10. I am missing any candidates' genes selected based on this transcriptome study. As authors mentions at the end of discussion, comparing the expression profiles with previously published GWAS or QTL mapping could narrow down number of candidates. I think including such a comparison in here would make manuscript stronger! I will be curios to see such compairison! |
| --- |

| **Attachment:** | [Reviewer 1 Review Attachment 1 - 07-14-2023 06:44:53](https://plantdirect.msubmit.net/pd_files/2023/06/15/00001450/00/1_reviewer_attachment_1_1688435675_convrt.pdf) |
| --- | --- |

**DECISION LETTER- Round 2**

July 18, 2023
Dr. Emmanuelle Graciet
University of Ireland Maynooth
Maynooth, N/A
Ireland


MSID: 2023-01245R1
MS TITLE: Transcriptional analysis in multiple barley varieties identifies signatures of waterlogging response

Dear Dr. Emmanuelle Graciet:

I am pleased to inform you that your manuscript "Transcriptional analysis in multiple barley varieties identifies signatures of waterlogging response" has been accepted for publication in Plant Direct.

Your article will appear online in the next available issue of Plant Direct. To ensure your article gets published as quickly as possible, please pay attention to the steps detailed below. We have found that most of the delays happen at this stage, especially at the payment stage, so please respond as quickly as possible when prompted.

License Agreement: Once your article has been accepted it will move to Production and undergo admin and file checking - you may receive an email with any queries we have at this stage. When all required items are received by the publisher and queries resolved, the corresponding author will receive an email from Wiley's Author Services system which will ask them to log on at https://authorservices.wiley.com/bauthor and will present them with the appropriate license for completion. Your article cannot be published until both the signed license agreement and payment of the article fee have been received.

Payment of the Open Access Article Publication Fee: All articles published in Plant Direct are fully open access: immediately and freely available to read, download and share. Plant Direct charges a publication fee to cover the publication costs. The corresponding author for this manuscript should have already received a quote from the payments team (cs-openaccess@wiley.com) with the estimated article publication fee; please email cs-openaccess@wiley.com if this has not been received. The corresponding author should log on to the Wiley Author Services site, where the publication fee can be paid by credit card or an invoice. Pro Forma can also be requested. Payment of the publication charge must be received before the article will be published online.

Proofs: You will have the opportunity to look over your paper once more when you receive the author proofs for your article. The proofs will be with you in approximately two weeks. Please note that, in addition to publishing reviewer comments, the author's responses to review comments will also be published alongside the final version of the paper. If you would not like the author's responses to be published, please contact the editorial office at plantdirect@wiley.com .

Promotion of your article: You can help your research get the attention it deserves! Check out Wiley's free Promotion Guide for best-practice recommendations for promoting your work at www.wileyauthors.com/eeo/guide. And learn more about Wiley Editing Services which offers professional video, design, and writing services to create shareable video abstracts, infographics, conference posters, lay summaries, and research news stories for your research at www.wileyauthors.com/eeo/promotion.

Thank you again for your contribution to Plant Direct. If you have any questions, feel free to contact the editorial office at plantdirect@wiley.com .

Sincerely,

Ana Fortes

Ana Fortes

Editor, Plant Direct


---------------------------------------------------------------------------- Editor comments


---------------------------------------------------------------------------- Reviewer comments:
Reviewer #2:

I thank the authors for providing additional data and analysis to address my comments. I recommend the revised article to be accepted.

**AUTHORS’ RESPONSE**

**Editor comments:**

*One of the reviewers raised important aspects regarding experimental design that should be fully addressed/ justified ( e.g. number of biological replicates used in transcriptomics analyses.)*

We have outlined more clearly the experimental design and what we meant by independent replicates. We used ‘independent replicates’ with the meaning of ‘biological replicates’. We have corrected this systematically in the manuscript, and provided more details in our response to the reviewers below.

**Reviewer #1:**

We wish to thank the reviewer for the comments received. We have addressed them as outlined below.

*The authors need to clarify what they mean when they state 'independent replicates' vs. technical and biological replicates. This was done in figure descriptions but should be clearly described in the methods.*

We apologize for this confusion, which was also highlighted by the 2^nd^ reviewer. We used ‘independent replicates’ with the meaning of ‘biological replicates’. We have corrected this systematically in the manuscript.

For the molecular experiments presented (RNA-seq and RT-qPCRs), in each biological replicate, we pooled roots of 3 plants. We repeated the experiments independently at least 3 times, as indicated in the figure legends. In the section where we describe the RNA extraction method, we have added “*For each condition (waterlogged or untreated), the root systems of 3 plants of the same variety were pooled prior to grinding for total RNA extraction. This experiment was conducted independently at least 3 times to obtain samples from at least 3 biological replicates, as indicated in the figure legends.*”.

For the physiological experiments, we took measurements from all of the plants we had in each biological replicate. We carried out the number of biological replicates indicated in the figure legends.

*Line 204: Please note the company or organization used to obtain Golden Promise seeds or make it clear that this variety came from the same source as the others.*

We obtained Golden Promise seeds from Teagasc and have indicated this in the materials and methods section “*(obtained from Teagasc, Oak Park, Ireland)*”. We also specified this for *Infinity*.

*Line 262: Was only a single replicate tested for NGS? The authors state that the roots of three different biological replicates were pooled. This would result in a strong single pool but only a single biological replicate. Was this pooling then repeated three times for additional biological replication? Please clarify.*

We apologize for the confusion: we sequenced sets of total RNA that originated from 3 biological replicates, which were carried out completely independently of each other. For each of these biological replicates, we pooled roots of 3 plants of the same variety and for one given experimental condition (control or waterlogged). This pooled tissue was used for RNA extraction and constituted the sample for one biological replicate. The experiments were repeated separately 3 times until we had samples from 3 biological replicates.

We have now indicated the following in the materials and methods section where we describe the RNA-seq experiment:

“*For RNA-seq analysis, waterlogging treatment was applied as outlined above for 24 h. For each condition (waterlogged or untreated), the root systems of 3 plants of the same variety were pooled prior to grinding for total RNA extraction. This experiment was conducted independently 3 times to obtain samples from 3 biological replicates (i.e. for each variety, 6 RNA samples were sent for sequencing, corresponding to 3 biological replicates for the untreated plants and 3 biological replicates for the waterlogged plants).*”

*Line 301: Missing year for citation.* Year added in citation *Line 304: Missing year for citation.*Year added in citation *Line 633: Missing page number.*Citation corrected and page number added. The unusual format for this reference is due to the fact that this is an online publication. *Line 649: Missing page number*. Citation corrected and page number added. The unusual format for this reference is due to the fact that this is an online publication. *Line 659: Missing page number.*Citation corrected and page number added*.* The unusual format for this reference is due to the fact that this is an online publication. *Line 670: Missing page number.* Citation corrected and page number added*.* The unusual format for this reference is due to the fact that this is an online publication. *Line 680: Missing page number.* Citation corrected and page number added*.* The unusual format for this reference is due to the fact that this is an online publication. *Line 733: Missing page number.* Citation corrected and page number added*.* The unusual format for this reference is due to the fact that this is an online publication. *Line 739: Bold volume number.*This citation is as requested by the authors of the Morex genome version 3. We have added a hyperlink for completeness*.
Line 772: Missing page number.* Citation corrected and page number added*.* The unusual format for this reference is due to the fact that this is an online publication.

*Figure 2: What do you mean by "independent replicates" here? Did you pool samples for measurements or average data from all roots/ shoots of each sample?*

As outlined above, for the physiological experiments, we took measurements from all the plants we had in one biological replicate. We repeated the experiments at least 3 times independently/separately (biological replicates), as indicated in the figure legends. We have corrected the figure legends to make the number of biological replicates clearer.

*Figure 5A: This figure could be improved by adding the gene names beside the heat map. This is cumbersome but very useful to decipher the exact genes altered in each group. 
The first citation for Supplemental Data is missing the page number and the last citation uses a slightly different format that the rest. Please correct.*

We agree with the reviewer that it would be nicer to indicate the gene names in Fig. 5A, so the reader can identify quickly the genes in question. Unfortunately, considering the number of genes represented (10,882 DEGs), we have found it difficult to generate a figure that would be legible. The genes present in each of the clusters are indicated in the Supplemental Table S4. We feel that this is a clearer manner to present the data to the readers.

We added the page number for the first reference in the Supplemental Information file. The unusual format for the last reference is due to the fact that this is an online publication.

**Reviewer #2:**

We thank the reviewer for the comments received. We have tried to address them as outlined below.

*1. HvACTIN was selected as reference gene based on its stable expression under control and waterlogged conditions over time. However, expression is shown only in one variety (Fig. S1)? Did authors make sure this gene is expressed to the same levels in few more varieties? I think it is essential to test it.*

When analyzing our RT-qPCR data, we have indeed checked that the level of expression of Hv*ACTIN* did not change between control and waterlogged samples, and that there was little variation between varieties. We now provide the data in a revised Fig. S1C (see relevant panel below). Please note that the slightly higher variation for *Arma*, *Louise*, *Isa* and *Retriever* is due to variation between replicates, as opposed to changes in the expression of Hv*ACTIN* between control and waterlogged samples.


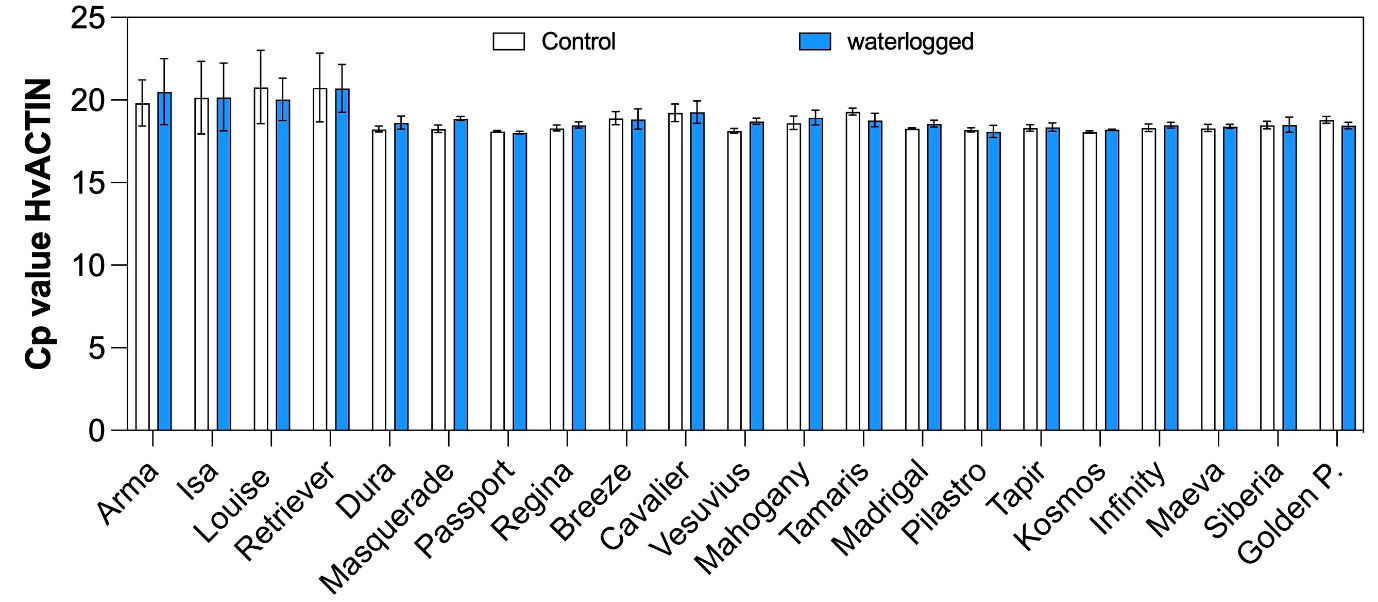


*Mean Cp values for HvACTIN for all varieties and RT-qPCRs presented in Fig. 1. Error bars correspond to SEM.*

*2. The method section should clarify number of replicates used for transcriptome analysis (considering that 3 root system were pooled as one replicate), as it is done in the figure 1*

We apologize for the confusion: we sequenced sets of total RNA that originated from 3 biological replicates, which were carried out completely independently of each other. For each of these biological replicates, we pooled roots of 3 plants. This pooled tissue was used for RNA extraction and constituted the sample for one biological replicate. The experiments were repeated separately 3 times until we had samples from 3 biological replicates.

We have now indicated the following in the materials and methods section where we describe the RNA-seq experiment:

“*For RNA-seq analysis, waterlogging treatment was applied as outlined above for 24 h. For each condition (waterlogged or untreated), the root systems of 3 plants of the same variety were pooled prior to grinding for total RNA extraction. This experiment was conducted independently 3 times to obtain samples from 3 biological replicates (i.e. for each variety, 6 RNA samples were sent for sequencing, corresponding to 3 biological replicates for the untreated plants and 3 biological replicates for the waterlogged plants).*”

*3. Figure 1 mentions: "Note: expression values for Infinity shown in (B) and (C) were published in (Miricescu et al., 2021)." Were the conditions of this study the same as presented here? If values for this variety comes from independent experiment, I would suggest to plot them separately.*

The samples for *Infinity* in Figure 1 were obtained in the same biological replicates as those for the other varieties. When we published our previous paper (Miricescu et al 2021), the reviewers requested the addition of RT-qPCR data, so we used the data we had generated in this larger experiment and published them on their own. It is only while writing this manuscript that we realized the need to publish the data as part of the full set. We added the note in the figure legend to ensure full transparency.

*4. Figure 2. I am again confused about the number of replicates. Do authors refer to independent experiments as independent replicates? The use of colors and shapes is not easy to follow. The shapes in the crowd of datapoints are hard to notice -I would suggest using two colors to mark control and waterlogging conditions. The colors indicating individual replicates (in my understanding independent experiments, but please clarify) are also hard to distinguish. If the authors aimed to test its effect I would rather suggest to analyze the data with a mixed model, where experimental trail is included as an independent factor.*

Again, we apologize for this confusion, which was also highlighted by the 1^st^ reviewer. We used ‘independent replicates’ with the meaning of ‘biological replicates’. We have corrected this systematically in the manuscript.

We wished to present the data for individual plants in each of the biological replicates, so that the readers and reviewers may see the data distribution and reproducibility across replicates more easily. To address the reviewer’s comment, we have represented the untreated samples in grey and the waterlogged samples in blue, and have removed the color coding of each of the biological replicates.

*5. In figure 3 I suggest using different colors and shadings (or other visualization) to indicate other variables, like variety, 2 vs 6 row and winter vs spring variety.*

We have tried to provide additional information as suggested by the reviewer, but it makes the grouping of the varieties difficult to see. We would prefer to keep this figure as it was presented in the first submission. We hope that the additional panels provided in the supplement (Fig. S3) will help the readers visualize other possible groupings according to the other variables mentioned by the reviewer.

*6. Figure 3C, Figure S4. Colors used are confusing, I would suggest using same color for each genotype and denote conditions by shading or intensity.*

We have made the requested changes in these figures. We used the same colour for each variety and made the control samples paler than the waterlogged samples to differentiate between treatments.

*7. In line 422, the authors refer to several Arabidopsis homologs in barley. Is the function of the genes also conserved (are they orthologs?). Do authors have phylogenetic trees supporting this relationships?*

We have not carried out phylogenetic analyses for the different homologs mentioned and hence prefer not to use the term of ‘ortholog’. However, for a more neutral term, we would happy to use ‘sequelog’ if the reviewer prefers it. This term was coined by Prof. Alexander Varshavsky to refer to genes (or proteins) that share sequence similarities without inferring a functional similarity (*'Spalog' and 'sequelog': neutral terms for spatial and sequence similarity*; [10.1016/j.cub.2004.02.014](https://doi.org/10.1016/j.cub.2004.02.014)).

*8. For the heatmap in figure 5 I would suggest clustering the columns as well -this would allow to see responses of which varieties are similar. I would also love to see it with the relation to the phenotypic observations, maybe by highlighting Regina as the one with least response in root and shoot growth?*

We have applied clustering to the columns, as suggested by the reviewer (see figure below). However, we do not find any obvious correlation with the phenotypic observations. If there are correlations, it seems to be with the number of DEGs identified in each of the varieties. For example, samples from waterlogged *Regina* and *Infinity* cluster apart from their respective controls (compared to other varieties), and this appears to correlate with the higher number of DEGs in *Regina* and *Infinity*.


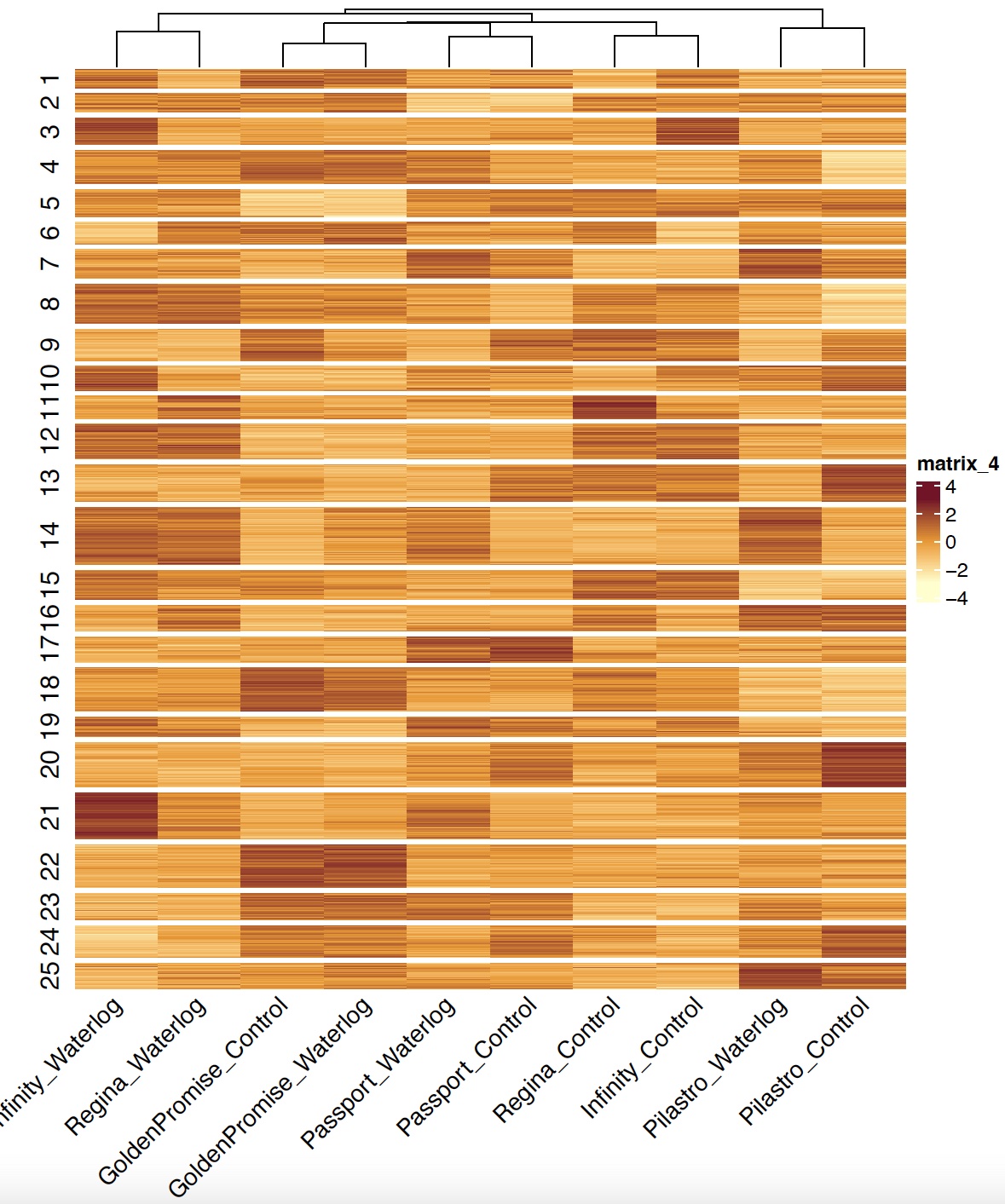


*9. What was the p-value threshold fr the GO enrichment analysis?*

The GO enrichment analyses were performed using ShinyGO and a threshold for the False Discovery Rate (FDR) of 0.05.

*10. I am missing any candidates' genes selected based on this transcriptome study. As authors mentions at the end of discussion, comparing the expression profiles with previously published GWAS or QTL mapping could narrow down number of candidates. I think including such a comparison in here would make manuscript stronger! I will be curios to see such compairison!*

We agree with the reviewer and have therefore compared our datasets to genes associated with QTLs for waterlogging tolerance in barley. This analysis allows us to pinpoint a couple of genes that could be prioritized for future studies. We have added a paragraph in the Methods section to describe this analysis and we have included the results in Supp. Table 4. We have also included text at the end of the Discussion to present the results of this analysis and a short discussion of the findings:

“*For example, we compared our data to a list of 28 genes located in QTLs for waterlogging tolerance (Supp. Table S4) that were identified following a screen of nearly 700 barley varieties with a specific focus on root traits (i.e. the formation of adventitious roots and of root cortical aerenchyma) (Manik et al., 2022). A number of candidate genes that are a part of this list are differentially regulated between the control samples or the waterlogged samples of the different varieties we tested. These genes include the homolog of HRE2 (HORVU.MOREX.r3.6HG0621670) – one of the ERFVII transcription factors that regulates the hypoxia response program, as well as a potassium transporter (HORVU.MOREX.r3.7HG0736590). This gene may be relevant because regulation of potassium flux during waterlogging has been shown to be important (Gill et al., 2018). Although the expression does not necessarily change in response to waterlogging, the intrinsic expression differences between varieties may be of interest to breeding waterlogging tolerant varieties in barley.”*
